# From protocol to published report: a study of consistency in the reporting of academic drug trials

**DOI:** 10.1186/s13063-016-1189-4

**Published:** 2016-02-19

**Authors:** Louise Berendt, Torbjörn Callréus, Lene Grejs Petersen, Karin Friis Bach, Henrik Enghusen Poulsen, Kim Dalhoff

**Affiliations:** The GCP Unit at Copenhagen University Hospital, Bispebjerg University Hospital, Copenhagen, Denmark; Medicines Development & Clinical Trials, Danish Health and Medicines Authority, Copenhagen, Denmark; Department of Clinical Pharmacology, Bispebjerg University Hospital, Copenhagen, Denmark; Laboratory of Clinical Pharmacology, Rigshospitalet, Denmark; Faculty of Health and Medical Sciences, University of Copenhagen, Copenhagen, Denmark; Present affiliation: Novo Nordisk A/S, Copenhagen, Denmark; Present affiliation: Division of Pharmacovigilance and Medical Devices, Danish Health and Medicines Authority, Copenhagen, Denmark; Present affiliation: Faculty of Health Sciences, University of Copenhagen, Copenhagen, Denmark; Nybro Vænge 3, Kongens Lyngby, DK-2800 Denmark

**Keywords:** Clinical trial methodology, outcome-reporting bias, academic research, drug research, clinical trials, published report

## Abstract

**Background:**

Unacknowledged inconsistencies in the reporting of clinical trials undermine the validity of the results of the trials. Little is known about inconsistency in the reporting of academic clinical drug trials. Therefore, we investigated the prevalence of consistency between protocols and published reports of academic clinical drug trials.

**Methods:**

A comparison was made between study protocols and their corresponding published reports. We assessed the overall consistency, which was defined as the absence of discrepancy regarding study type (categorized as either exploratory or confirmatory), primary objective, primary endpoint, and – for confirmatory trials only – hypothesis and sample size calculation. We used logistic regression, χ^2^, and Fisher’s exact test.

**Results:**

A total of 282 applications of academic clinical drug trials were submitted to the Danish Health and Medicines Authority in 1999, 2001, and 2003, 95 of which fulfilled the eligibility criteria and had at least one corresponding published report reporting data on trial subjects. Overall consistency was observed in 39 % of the trials (95 % CI: 29 to 49 %). Randomized controlled trials (RCTs) constituted 72 % (95 % CI: 63 to 81 %) of the sample, and 87 % (95 % CI: 80 to 94 %) of the trials were hospital based.

**Conclusions:**

Overall consistency between protocols and their corresponding published reports was low. Motivators for the inconsistencies are unknown but do not seem restricted to economic incentives.

## Background

The obligation to make results of clinical trials available to the public is stated in the Declaration of Helsinki [1]. Furthermore, the validity of trial conclusions depends on the use of stringent scientific methods as well as transparency in the reporting of the results.

Unacknowledged discrepancies between protocols and their corresponding published reports may undermine the validity of the scientific effort [2], produce unfounded conclusions, and lead to the unnecessary repetition of trials with identical hypotheses and loss of generated knowledge [3]. Particularly in the case of large long-lasting clinical trials that are unlikely to be repeated, such inconsistencies may jeopardize the risk-benefit ratio of the investigational drug. At the regulatory level, changes made during the conduct of a trial may invalidate the risk-benefit assessment that lead to the initial approval of the trial.

A recent Cochrane review [4] found that published reports of randomized controlled clinical trials (RCTs) frequently differ from their corresponding protocols or trial registry data, for example, in the primary outcomes [5–11] and sample size calculations [7, 8, 12, 13] for interventions such as surgery, cosmetics, drugs, and healthcare counseling. Discrepancies have also been found in the reporting of drug trials [10, 13–18]. Previous results mainly reflect the reporting of commercial trials (range: 61 % to 100 % of study samples [7, 12, 14, 17, 19, 20]) or highly selected cohorts of publicly funded phase III oncology trials and HIV RCTs [13, 15, 21]. However, all reviews indicated similar problems. Discrepancies have also been found in published reports of government-funded RCTs of various clinical specialties [8]. Whether these extend to non-commercial drug trials, called academic drug trials, is unknown. These trials are unrelated to drug companies or similar economic influences and conducted in an array of clinical specialties. We therefore investigated the prevalence of the consistency between protocols and corresponding published reports of Danish academic clinical drug trials.

## Methods

The study sample consisted of all approved academic clinical drug trial applications submitted to the Danish Health and Medicines Authority in 1999, 2001, and 2003, which has been described previously [22]. The trials were defined on the basis of the data as well as the publication rights being the property of publicly employed researchers and the absence of a pharmaceutical company name on the first page of the protocol. Trials with the sponsor living outside Denmark were excluded, whereas 39 previously examined trials were included [23].

### Screening

For each trial, the corresponding published reports were identified from May to September 2009 by a systematic PubMed search. The follow-up time was at least 5 years. The search terms were based on selected data from electronic files at the Danish Health and Medicines Authority: sponsor’s name, protocol title, investigational medicinal products, and a brief description, if available, of the study. A published report was defined as any article reporting data on the trial subjects. PhD theses, conference abstracts, reviews, and published reports not reporting data on trial subjects were excluded as these are either not indexed, not sufficiently detailed, or do not contain trial results.

The names of the submitting sponsors were extracted from all included trials, and contact information was updated by searching Google or a registry of Danish physicians. Contact to sponsor was made by e-mail or letter for confirmation or correction of the identified corresponding published report(s) or lack thereof. Two reminders were sent in case of no response.

### Data collection

Data were extracted from the protocols, including correspondence and amendments, and from the corresponding published reports. Pre-specified definitions of consistency and discrepancy of the composite variables were developed and tested. Data were extracted by LB, and uncertainties were discussed with LGP and TC. A continuous decision log ensured reproducibility.

To avoid confusion, *main outcomes* denote those of our study, whereas *primary endpoints* denote those of the trials in the study sample. The main outcome was overall consistency between protocols and their corresponding published reports, which was a priori defined as consistency on all of the following variables: study type (exploratory/confirmatory), primary objective and primary endpoint and – for pairs of confirmatory protocols and corresponding confirmatory published reports – also as consistency in the hypothesis and sample size calculation. We also calculated the number of discrepancies per trial and the prevalence of discrepancy regarding each of the component variables.

If a published report showed discrepancy on a given variable but provided transparency, by either clearly stating the deviation from the protocol or referencing a previous published report that describes the study in accordance with the protocol, the variable was considered consistent.

The variables were defined as indicated below.

#### Discrepancy in the study type

We defined a confirmatory protocol/published report as a study testing a pre-specified hypothesis, which was associated with a formal sample size calculation. Studies with a primary confirmatory analysis and secondary exploratory analyses were considered confirmatory. All other studies were considered exploratory. A published report was categorized as discrepant if the study type differed from the study type derived from the protocol.

#### Discrepancy in the primary objective

The primary objective was defined as an objective explicitly defined as such. If there was no explicitly defined primary objective, the objective related to the primary endpoint was considered primary. In the special case of protocols consisting of more than one explicitly defined primary objective, consistency was determined as follows: 1) A published report stating the same or some of the protocol-specified primary objectives was considered consistent with the protocol. 2) A published report stating a non-protocol-specified primary objective was considered discrepant regardless of the consistency of other primary objectives. A published report only reporting secondary objectives and not stating the protocol-specified primary objective was considered consistent with the protocol only if a published report reporting or stating the primary objective was referenced (that is, providing transparency in the published report).

#### Consistency in the primary endpoint

The primary endpoint(s) was (were) defined as the one or two endpoints that were explicitly defined as primary. If no primary endpoint was explicitly defined, the endpoint used in the sample size calculation was considered as primary. If more than two endpoints were explicitly defined as primary, the protocol was considered to have no primary endpoints. In case of within-protocol or within-published report inconsistency, only the primary endpoint(s) substantiated in the body text was considered as primary. If one of two published report-specified primary endpoints differed from the primary endpoint(s) specified in the protocol, the published report was considered discrepant.

Pairs of confirmative protocol/confirmative published reports were also reviewed regarding discrepancy in the hypothesis and sample size calculation.

#### Discrepancy in the hypothesis

Hypotheses from the protocol and published report were compared. In the absence of an explicitly defined hypothesis, we formulated a hypothesis based on the sample size calculation as well as the rationale of the study (for example, “A better than B”). In case of a within-protocol inconsistency, the formulated hypothesis was based on the sample size calculation. For example, a protocol with a research question suited for an equivalence or noninferiority trial, but statistically designed to demonstrate superiority, was considered a superiority trial.

#### Discrepancy in the sample size calculation

The sample size calculation was considered discrepant if either the calculated sample size or any of the available components from the calculation differed between the protocol and the published report. It was also considered as a discrepancy if a sample size calculation was stated in the protocol but missing from the published report. The achieved sample size was not taken into account.

### Data analysis and statistics

The sample size calculation was based on expected frequencies of overall consistency of 40 or 62 % of the trials. A sample size of 100 trials was chosen because the inclusion of at least 92 trials would yield a standard error of proportion (SEP)*z_2α_ less than 0.1. Data were registered in a Microsoft Access database with audit trail and analyzed in SAS 9.2 using χ^2^ and Fischer’s exact tests and logistic regression. *P* values < 0.05 were considered statistically significant. Kappa values were analyzed with GraphPad QuickCalcs (http://graphpad.com/quickcalcs). Multivariate logistic regression was planned but not conducted because only a few of the pre-specified variables for the regression showed association with overall consistency in 2 × 2 tables. We conducted post hoc logistic regression analyses adjusted by the association between published reports of the same protocol. This was done by the use of a repeated measures statement and with published reports as the unit of analysis.

Intra-rater agreement during data collection was determined from the test-retest of five protocols and 16 corresponding published reports assessed within an interval of 6 months. The variables were assumed independent of each other. Study types, primary objectives, and primary endpoints were extracted from 21 documents (five protocols and 16 published reports). Hypotheses and sample sizes were extracted from 10 documents (four protocols and six published reports). Overall, 77 of the 83 data points showed perfect agreement. The six disagreements were distributed as follows: primary endpoint (2/10), hypothesis (1/10), primary objective (1/10), and sample size calculation (2/10). No disagreements were found regarding trial type (exploratory/confirmatory).

## Results

**Fig. 1:**
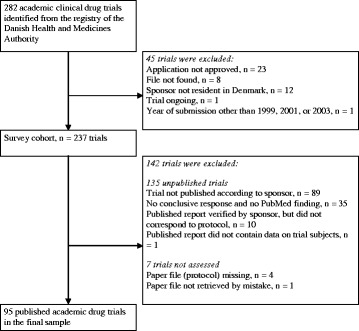
Flow chart

A total of 282 applications of academic drug trials were submitted to the Danish Health and Medicines Authority in the period, 117 of which had more than one corresponding published report and were included for assessment (Fig. 1). During data collection, 22 trials were excluded for the following reasons: investigator not a resident in Denmark (*n* = 10), published report did not correspond to the protocol (*n* = 10, in a few cases, despite investigators verification), the published report contained no data on trial subjects (*n* = 1), or the year of application other than 1999, 2001, or 2003 (*n* = 1). The minimum follow-up time from approval of protocol to screening publication rate was 40 % (95/237). The final sample consisted of 95 approved clinical drug trials comprising 95 protocols and 143 corresponding published reports (median: one published report per trial, range: one to eight). Of those, 42 (46 %) protocols described an exploratory trial, whereas the remaining 53 (54 %) were confirmatory. Characteristics of the exploratory and confirmatory protocols are shown in Table 1, and characteristics of the subgroup of controlled exploratory and confirmatory trials in Table 2.

**Table 1:** Protocol characteristics of academic clinical drug trials by type of study

		Type of study		
	Total *n* (%)	Exploratory *n* (%*)	Confirmatory *n* (%*)	*P*
Number of trials	95	42	53	-
Single/multicenter				
Single center	71 (75 %)	31 (74 %)	40 (75 %)	0.85
Multicenter, national	16 (17 %)	8 (19 %)	8 (15 %)	0.78
Multicenter, international	8 (8 %)	3 (7 %)	5 (9 %)	1.00^**^
Sponsor’s affiliation				
Hospital	83 (87 %)	40 (95 %)	43 (81 %)	0.06^**^
Medical practice	8 (8 %)	0 (0 %)	8 (15 %)	0.01^**^
University or other	4 (4 %)	2 (4 %)	2 (4 %)	1.00^**^
Good clinical practice (GCP)				
Adherence to GCP	33 (35 %)	16/41 (39 %)	17/53 (32 %)	0.48
Missing (not stated in protocol or application)	1 (1 %)	1 (2 %)	0 (0 %)	1.00^**^
GCP monitoring	27 (28 %)	12 (29 %)	15 (28 %)	0.89
Control of compliance	29/51^#^ (57 %)	8/15^#^ (53 %)	21/36^#^ (58 %)	0.86
Control groups				
Control group	76 (80 %)	26 (62 %)	50 (94 %)	<0.001
Control group, historical	0 (0 %)	0 (0 %)	0 (0 %)	-
Endpoints, power and sample size				
Statement of endpoints	95 (100 %)	42 (100 %)	53 (100 %)	-
Statement of one to two primary endpoints	73 (77 %)	23 (55 %)	50 (94 %)	<0.001^**^
Statement of sample size calculation	65 (68 %)	12 (29 %)	53 (100 %)	<0.001^**^
Statement of statistical methods	72 (76 %)	32 (76 %)	40 (75 %)	0.94
Funding				
External funding granted or wanted	61/83 (73 %)	27/38 (71 %)	34/45 (76 %)	0.64
Missing (not stated in protocol or application)	12 (13 %)	4 (10 %)	8 (15 %)	0.54^**^

*Prevalence in each subgroup, for example, a control group is present in 26 of 42 exploratory trials (62 %)

**Fischer’s exact test due to fewer than five observations in a single cell

^#^ *n* = 51, because compliance was considered “not relevant” in 27 exploratory and in 17 confirmatory trials

**Table 2:** Protocol characteristics of academic clinical drug trials by type of study, continued

		Type of study		
	Total *n* (%)	Exploratory *n* (%*)	Confirmatory *n* (%*)	*P*
Number of controlled trials	76	26	50	-
Design				
Parallel	38 (50 %)	11 (42 %)	27 (54 %)	0.33
Cross-over	37 (49 %)	14 (54 %)	23 (46 %)	0.52
Other	1 (1 %)	1 (4 %)	0 (0 %)	0.34^**^
Blinding	57 (75 %)	16 (62 %)	41 (82 %)	0.051
Randomization	69 (91 %)	20 (77 %)	49 (98 %)	0.006

*Prevalence in each subgroup; for example, a parallel design was used in 27 of the confirmatory trials (54 %)

Most of the trials, 73 % (95 % CI: 63 to 81 %), were randomized controlled trials (RCTs). The majority had one or two pre-specified primary endpoints (77 %, 95 % CI: 68 to 85) and pre-specified statistical methods (76 %, 95 % CI: 67 to 84 %). Most sponsors were employed in hospitals within the Capital Region of Denmark (59 %, 95 % CI: 49 to 69 %) and were receiving, applying for, or going to apply for grants from external sources (73 %, 95 % CI: 64 to 83 %).

Overall consistency was observed in 39 % of the trials (95 % CI: 29 to 49 %, Table 3). The frequency was lower among confirmatory trials compared to exploratory trials (30 % versus 50 %). In comparison, overall consistency was observed in 49 % of the published reports (95 % CI: 41 to 57 %). Confirmatory published reports were less likely to show overall consistency compared to exploratory published reports (adjusted OR 0.37, 95 % CI: 0.17 to 0.83, Table 4).

**Table 3:** Overall consistency and discrepancy between protocols and corresponding published reports of academic drug trials by protocol-derived type of study, *N* = 95 protocols

	All trials		Exploratory trials		Confirmatory trials	
	*n*	% (95 % CI)	*n*	% (95 % CI)^*^	*n*	% (95 % CI)^*^
Number of trials	95		42		53	
Overall consistency	37	39 % (29 to 49 %)	21	50 % (35 to 65 %)	16	30 % (18 to 43 %)
Individual discrepancies						
Type of study	22	23 % (15 to 32 %)	6	14 % (4 to 25 %)	16	30 % (18 to 43 %)
Primary objective	19	20 % (12 to 28 %)	12	29 % (15 to 42 %)	7	13 % (4 to 22 %)
Primary endpoint	39	41 % (31 to 51 %)	16	38 % (23 to 53 %)	23	43 % (30 to 57 %)
Hypothesis	- 17	-	-	-	5/37^◊^	14 % (2 to 25 %)
Sample size calculation	-	-	-	-	17/37^◊^	46 % (30 to 62 %)

*Prevalence in each subgroup; for example, 21 of 42 exploratory trials (50 %) showed overall consistency

^◊^Based on the subgroup of 37 trials with a confirmatory protocol and more than one confirmatory published report

**Table 4:** Overall consistency and discrepancy between protocols and corresponding published reports by published report-derived type of study, N = 143 published reports

	Exploratory published reports *N* = 96		Confirmatory published reports *N* = 47		Crude OR	Adjusted OR^#^
	*n*	% (95 % CI)	n	% (95 % CI)	OR (95 % CI)	OR (95 % CI)
Overall consistency	53	55 % (45 to 65 %)	17	36 % (22 to 50 %)	0.46 (0.22 to 0.94)	0.37 (0.17 to 0.83)
Individual discrepancies						
Type of study	17	18 % (10 to 25 %)	6	13 % (3 to 22 %)	0.68 (0.25 to 1.87)	0.64 (0.21 to 1.48)
Primary objective	21	22 % (14 to 30 %)	5	11 % (2 to 19 %)	0.43 (0.15 to 1.21)	0.56 (0.11 to 2.80)
Primary endpoint	34	35 % (26 to 45 %)	13	28 % (15 to 40 %)	0.70 (0.32 to 1.50)	0.68 (0.31 to 1.48)

*Prevalence in each subgroup; for example, 53 of 96 exploratory published reports (55 %) showed overall consistency

^#^Adjusted for the association between published reports of the same trial (median: one published report per trial, range: one to eight)

The individual discrepancies are shown in Tables 3 and 4. The most prevalent was the primary endpoint discrepancy (41 % of the trials, 95 % CI: 31 to 51 %). Similarly, primary endpoint discrepancy was observed in 33 % of the published reports (95 % CI: 25 to 41 %). In neither of the analyses did the occurrence of primary endpoint discrepancy seem to be associated with the study type (exploratory or confirmatory).

Of the 58 trials with at least one discrepancy, 23 trials were associated with one discrepancy, and 35 trials with two or more discrepancies. Half of the published reports (73/143) showed discrepancy. Of these 73 published reports, 35 had one discrepancy, 31 had two, and seven had three discrepancies. None had more than three discrepancies.

Agreement on the published report status between the survey and the literature search was estimated from the 183 trials with a conclusive survey response. The Kappa statistic κ = 0.782 indicated good agreement (95 % CI: 0.692 to 0.872, agreement for 91 % of the trials).

## Discussion

In this review and follow-up of academic drug trials in Denmark, we found overall consistency between the approved protocol and resulting published reports in 39 % (95 % CI: 51 to 70 %) of the trials. The assessment of overall consistency included the following composite variables: primary objective, primary endpoint, type of study, hypothesis, and reporting of power calculation. The most prevalent was discrepancy on the primary endpoint (41 %), but the type of study and primary objective differed frequently as well, among 23 % and 20 % of the trials, respectively. The publication rate of 40 % is comparable to our findings in a similar cohort (33 %) [22].

Few studies of the reporting of clinical trials have been conducted on academic drug trials [13, 15, 21] or even academic drug/non-drug trials [9]. To our knowledge, this is the first investigation of protocol-published report consistency in academic drug trials across medical specialties. To provide data as solid as possible, we used predefined eligibility criteria based on the ownership of trial data and publication rights rather than the source(s) of funding, we included RCTs and non-RCTs from all medical specialties, and we categorized the trials by the nature of their research question as exploratory or confirmatory. Furthermore, we constructed a composite outcome, overall consistency, which took into account some of the differences between exploratory and confirmatory trials. The bias from selection of trials was minimized because we had access to all trials approved in Denmark.

Previous studies on the reporting of drug trials [10, 13–18] primarily included commercial trials. However, our study demonstrates that drug trials that cannot be assumed to involve heavy economic interests also have a similar lack of consistency. This is in agreement with the findings of Chan et al. [8] in a cohort of government-funded drug/non-drug RCTs. The observed discrepancies are of such a magnitude that we believe our results represent a real problem in the reporting of academic clinical drug trials. Previously, the focus has primarily been on RCTs, which require a well-defined design for the testing of specific hypotheses [24]. However, control groups and randomization are also used in exploratory trials. In our study sample, 20/69 (29 %) of the randomized trials were categorized as exploratory, whereas confirmatory studies constituted 4/26 (15 %) of the nonrandomized trials. This suggests that limitations apply to the use of randomization as an inclusion criterion in the evaluation of methodological quality from a perspective of evidence-based medicine.

To ensure consistency and reproducibility from using a single assessor (LB), we implemented quality assurance and control measures, such as the development and test of clear definitions of consistency and discrepancy, the keeping of a decision log during data collection, and the test-retest of the assessment of the outcome variables. The intra-rater agreement was assessed and reported using the pre-specified unit of measurement and analysis plan. Alternatively, intra-rater agreement could have been analyzed using protocols as the unit of measurement. This would have required a larger sample for the assessment but would also have provided a better estimate.

In a few cases, difficulties in collecting data from the protocols was due to the contradictory definitions of the primary endpoint, which points to other problems in the writing of the protocol as well as in the approval procedures of the competent authorities. Since this was not predefined in our study, we did not investigate it further.

Discrepancies within a published report or between published reports of the same trial may be associated. The adjusted and unadjusted post-hoc logistic regressions at the published report level did not change our primary results. Discrepancies were frequent in published reports regardless of the study type of the underlying protocol (exploratory/confirmatory) and the study type derived from the published report. We found a discrepancy in half of the 143 published reports, each carrying a risk of the study results being misinterpreted due to inadequate or misleading information. We did not assess whether the discrepancies were associated with the direction of the trial results. Nevertheless, the risk of bias exists, which may be forwarded to future research and clinical decisions.

We have not found previous reports on the transformation of exploratory protocols to confirmatory published reports, a topic that is highly important because the study type implies certain strengths and limitations to the interpretation of the study results [25, 26]. Such discrepancy in 14 % and 30 % of exploratory and confirmatory trials is problematic. Similarly, the discrepancy in primary endpoints in 41 % of academic clinical drug trials is critical but consistent with earlier findings (33 % to 62 % of RCTs [7, 8, 10]). Discrepancy regarding the primary objective was less frequent but was associated with discrepancy of the primary endpoint (*P* < 0.0001, χ^2^, data not shown).

Published reports neither stating nor referencing the protocol-specified (or any other) sample size calculation, and therefore defined as exploratory, were found in 46 % of the confirmatory trials; this figure is comparable to the 53 % reported by Chan [20] and the 59 % by Mhaskar [15]. The transparent reporting of the sample size calculation allows the reader to assess the power and pre-specified relevant clinical benefit of the study [27].

We studied a cohort of trials approved until 2003. Since then, the Clinical Trials Directive has been introduced, and the International Committee of Medical Journal Editors (ICMJE) has facilitated transparency in the reporting of research by requiring clinical trials to be registered in a publicly available database [28]. As of 2011, information on all clinical drug trials approved since May 2004 by the European medicines authorities is publicly available [29, 30]. The information is limited but is directly uploaded by the authorities at the time of approval, thus serving to reduce the problem of retrospective registration. Such resources provide a valuable tool to journal editors and reviewers if kept up to date with accurate information and if used actively during journal review. However, the occurrence of missing or unclear registry data [31, 32] and many trials being registered retrospectively [33] substantiates the continued use of protocols as the primary source of trial characteristics [34]. Evidence of discrepancies, even between trial registries and published reports [4], indicate that a dedicated effort is still required by the researchers as well as the journal editors and reviewers to promote transparency in the reporting of clinical research. Studies assessing the consistency in the reporting of clinical trials should be conducted at regular intervals to ensure a continued improvement in the reporting.

In Denmark, academic trials constitute a third of clinical drug trials [22], with a high proportion of confirmatory trials providing a considerable contribution to the accumulation of clinical evidence. The impact on clinical practice of academic versus commercial trials is unknown, but differences may exist. Probably, commercial trials have their main impact on the registration of drugs, whereas academic drug trials probably have mostly other impacts.

The reasons for discrepancy between protocols and published reports are unknown and may be complex, but it seems there are other motivators than economic driving forces. Previous studies point to reasons such as a lack of clinical importance, lack of statistical significance, and unawareness of the consequences of not reporting all outcomes and protocol changes [8, 35].

## Conclusion

In this study, we predefined overall consistency between protocols and published reports as the primary focus and found it to be low for academic clinical drug trials. The discrepancies pose an invisible threat to the validity of trial conclusions. These results indicate a general need for improving the consistency between protocols and the resulting published reports, particularly regarding the definition of the primary endpoint and of the trial as exploratory or confirmatory. Further studies are needed to assess improvements in the reporting of clinical trials over time.

## Abbreviations

CI: confidence interval
GCP: good clinical practice
HIV: human immunodeficiency virus
ICMJE: International Committee of Medical Journal Editors
OR: odds ratio
RCT: randomized controlled clinical trial
SEP: standard error of proportion
